# Supplementary material for: Precision medicine in type 2 diabetes: targeting SGLT2 inhibitor treatment for kidney protection
Source: Diabetologia. 2025 Nov 9;69(2):374–85. doi: 10.1007/s00125-025-06577-2 (PMC12779664; doi:10.1007/s00125-025-06577-2)
Supplement: Supplementary file 1 — ESM (PDF 1.16 MB) [file 125_2025_6577_MOESM1_ESM.pdf]

ELECTRONIC SUPPLEMENTARY MATERIAL (ESM)

Item	Page
ESM Methods	2
ESM Table 1	3
ESM Table 2	6
ESM Table 3	9
ESM Table 4	10
ESM Figure 1	13
ESM Figure 2	14
ESM Figure 3	15
ESM Figure 4	16
ESM Figure 5	17
ESM Figure 6	19
ESM Figure 7	20
ESM Figure 8	21
ESM Figure 9	22
Mastermind Consortium	24

ESM Methods

The formula for the CKD-PC risk score formula for ≥50% eGFR decline/ESKD is as follows:

$$\frac{e^{(-4.941086+linear\ predictor)}}{1+e^{(-4.941086+linear\ predictor)}}$$

Where the linear predictor is defined as follows:

$$\begin{aligned}
&0.0794321 \times ((age\ [in\ years] - 60)/10) - 0.1141482 \times (presence\ of\ male\ sex\ [binary] - 0.5) \\
&- 0.0459708 \times ((eGFR\ [in\ mL/min/1.73m2] - 85)/5 + 0.4326073 \times \ln(uACR\ [in\ mg/g]\ /\ 10) \\
&+ 0.1917602 \times ((systolic\ blood\ pressure\ [in\ mmHg] - 130)\ /\ 20) \\
&+ 0.306044 \times use\ of\ antihypertensive\ medication\ [binary] - 0.0670274 \times ((systolic\ blood\ pressure\ [in\ mmHg] \\
&- 130)\ /\ 20 \times use\ of\ antihypertensive\ medication\ [binary]) + 0.9185919 \times (history\ of\ heart\ failure\ [binary] - 0.05) \\
&+ 0.2386131 \times (history\ of\ coronary\ artery\ disease\ [binary] - 0.15) + 0.2157927 \times bistory\ of\ atrial\ fibrillation\ [binary] \\
&+ 0.1122236 \times current\ smoker\ [binary] + 0.1327815 \times former\ smoker\ [binary] + 0.0154271 \times ((BMI\ [in\ kg/m2] \\
&- 30)\ /\ 5) + 0.1103419 \times (HbA1c\ [in\ \%] - 7) - 0.0460205 \times use\ of\ oral\ antihyperglycaemic\ medication\ [binary] \\
&+ 0.2571656 \times use\ of\ insulin\ [binary]
\end{aligned}$$

Of note, history of heart failure (0), history of heart failure (0) and use of oral antihyperglycaemic medication (1) were constant in this study population.

ESM Table 1. Target trial emulation protocol.

Protocol components	Target trial	Emulation
**Eligibility criteria**	**Inclusion**:<br>- Type 2 diabetes<br>- SGLT2-inhibitor naïve<br><br>**Exclusion**:<br>- eGFR <60 ml/min/1.73m^2 or need for renal replacement therapy (dialysis or kidney transplant).<br>- Urinary albumin creatinine ratio ≥30 mg/mmol.<br>- Prior history of atherosclerotic vascular disease (i.e. myocardial infarction, stroke, or peripheral vascular disease) or heart failure.<br>- Use of GLP1-receptor agonists.	**Inclusion**<br>- Type 2 diabetes<br>- SGLT2-inhibitor naïve<br>- Receiving first-ever prescription for an SGLT2-inhibitor, DPP4-inhibitor or sulfonylurea.<br><br>**Exclusion**:<br>- eGFR <60 ml/min/1.73m^2 or need for renal replacement therapy (dialysis or kidney transplant).<br>- Urinary albumin creatinine ratio ≥30 mg/mmol.<br>- Prior history of atherosclerotic vascular disease (i.e. myocardial infarction, stroke, or peripheral vascular disease) or heart failure.<br>- Use of GLP1-receptor agonists.<br>- Less than 91 days of registration data before treatment initiation.
**Treatment assignment**	Random assignment to one of the two treatment arms (SGLT2-inhibitors vs DPP4-inhibitors/sulfonylureas).	We assume random assignment to one of the two treatment arms (SGLT2-inhibitors vs DPP4-inhibitors/sulfonylureas) by applying overlap weighting based on predefined covariates.
**Treatment initiation**	Initiation of only one antihyperglycaemic medication (SGLT2-inhibitor, DPP4-inhibitor, or sulfonylurea) at the time of treatment assignment.	We consider the date of a first prescription for one antihyperglycaemic medication (SGLT2-inhibitor, DPP4-inhibitor, or sulfonylurea) as date of treatment initiation.

**Treatment strategy**	Prescription issued by primary care practice. Further additional treatment after a 3-month treatment initiation period is allowed as needed.	Prescription information identified from primary care records (Clinical Practice Research Datalink, CPRD). Further additional treatment after a 3-month treatment initiation period is allowed as needed.
**Follow-up period**	Follow-up starts at treatment initiation. Subjects are followed up until the earliest of: occurrence of outcome, a maximum of 3 years, or October 15, 2020.	Follow-up starts at treatment initiation. Subjects are followed up until the earliest of: occurrence of outcome, a maximum of 3 years, or October 15, 2020. Subjects in the DPP4-inhibitor/sulfonylurea arm are censored if starting an SGLT2-inhibitor or GLP1-receptor agonist.
**Outcomes**	Primary outcome:<br>- Kidney disease progression defined as a composite of a ≥50% decline in eGFR, end-stage kidney disease (requirement for renal replacement therapy or sustained eGFR <15 ml/min/1.73m^2), or death with kidney disease listed as cause of death.<br>Secondary outcomes:<br>- A similar composite of a ≥40% decline in eGFR or end-stage kidney disease<br>- Progression to uACR ≥30 mg/mmol<br>- Diabetic ketoacidosis<br>- Amputation<br>- Mycotic genital infection	Primary outcome:<br>- Kidney disease progression defined as a composite of a ≥50% decline in eGFR, end-stage kidney disease (requirement for renal replacement therapy or sustained eGFR <15 ml/min/1.73m^2), or death with kidney disease listed as cause of death.<br>Secondary outcomes:<br>- A similar composite of a ≥40% decline in eGFR or end-stage kidney disease<br>- Progression to uACR ≥30 mg/mmol<br>- Diabetic ketoacidosis<br>- Amputation<br>- Mycotic genital infection
**Causal contrasts of interest**	Average treatment effects in intention-to-treat analyses.	Average treatment effects in intention-to-treat analyses.

**Analysis plan**	- Cox proportional hazard models.<br>- Subgroup analyses with stratification by presence of low-level albuminuria (≥3 mg/mmol).	- Doubly-robust Cox proportional hazards models (adjusted for predefined covariates and weighted for overlap weights).<br>- Subgroup analyses with stratification by presence of low-level albuminuria (≥3 mg/mmol).<br>- Sensitivity analyses with weighting for inverse probability of treatment weights instead of overlap weights, and multivariable adjustment alone.<br>- Sensitivity analyses with DPP4-inhibitors and sulfonylureas treated as separate treatment arms, with subjects censored if starting any of the other study drugs, analysed as described above.

Abbreviations: SGLT2-inhibitors sodium-glucose cotransporter-2 inhibitors, DPP4-inhibitors dipeptidyl peptidase-4 inhibitors, GLP1-receptor agonists glucagon-like peptide-1 receptor agonists, eGFR estimated glomerular filtration rate.

ESM Table 2. Baseline characteristics by albuminuria status.

	uACR <3 mg/mmol (*n*=116,197)	uACR ≥3 mg/mmol (*n*=25,303)
Sociodemographic characteristics		
Age, years	58 ±11	57 ±11
Male sex	67,999 (59%)	15,977 (63%)
Ethnicity		
White	86,720 (75%)	17,478 (69%)
South Asian	16,435 (14%)	4,705 (19%)
Black	7047 (6%)	1647 (7%)
Mixed	1287 (1%)	319 (1%)
Other or unknown	4708 (4%)	1154 (5%)
Index of multiple deprivation, deciles		
1-2	20,045 (17%)	3750 (15%)
3-4	21,047 (18%)	4241 (17%)
5-6	22,224 (19%)	4756 (19%)
7-8	25,919 (22%)	5983 (24%)
9-10	26,961 (23%)	6573 (26%)
Laboratory and vital signs measurements		
BMI, kg/m^2	32 ±7	33 ±7
Systolic blood pressure, mmHg	132 ±13	135 ±14
Diastolic blood pressure, mmHg	78 ±9	79 ±9
Total cholesterol, mmol/l	4.4 ±1.1	4.5 ±1.2
HbA_{1c}, mmol/mol	75 ±18	79 ±19
HbA_{1c}, %	9.0 ±3.8	9.4 ±3.9
eGFR, ml/min/1.73m^2	95 ±14	97 ±15
uACR, mg/mmol	0.9 [0.6, 1.6]	6.1 [4.1, 10.5]
Comorbidities		
Diabetes duration at treatment start, years	6.8 [3.6, 11.0]	7.3 [3.8, 11.7]

Smoking status		
Non-smoker	62,311 (54%)	12,581 (50%)
Ex-smoker	36,257 (31%)	7816 (31%)
Current smoker	17,630 (15%)	4906 (19%)
Arterial hypertension	57,873 (50%)	14,736 (58%)
Atrial fibrillation	2620 (2%)	702 (3%)
Previous diabetic keto-acidosis	465 (0%)	153 (1%)
Previous mycotic genital infection	13,239 (11%)	2873 (11%)
Hospitalisation in previous year	21,563 (19%)	5189 (21%)
Medications		
Calendar year at baseline		
2013	13,826 (12%)	2837 (11%)
2014	14,795 (13%)	3241 (13%)
2015	16,762 (14%)	3576 (14%)
2016	15,121 (13%)	3231 (13%)
2017	15,210 (13%)	3359 (13%)
2018	15,813 (14%)	3495 (14%)
2019	15,901 (14%)	3587 (14%)
2020	8769 (8%)	1977 (8%)
Number of current glucose-lowering treatments		
1	11,897 (10%)	2154 (9%)
2	67,175 (58%)	13,626 (54%)
3+	37,125 (32%)	9523 (38%)
On statin	94,551 (81%)	20,954 (83%)
On insulin	5875 (5%)	1955 (8%)
On ACE-inhibitor or ARB	64,014 (55%)	18,667 (74%)
Treatment arm		
SGLT2-inhibitor	43,078 (37%)	10,018 (40%)

DPP4-inhibitor/sulfonylurea	73,119 (63%)	15,285 (60%)

Values for continuous variables are mean±SD or median (IQR); those for categorical variables are *n* (%)

Abbreviations: BMI body mass index, eGFR estimated glomerular filtration rate (calculated using the Chronic Kidney Disease Epidemiology Collaboration equation), uACR urinary albumin-creatinine ratio, ACE angiotensin-converting enzyme, ARB angiotensin-II receptor blocker, SGLT2-inhibitors sodium-glucose cotransporter-2 inhibitors, DPP4-inhibitors dipeptidyl peptidase-4 inhibitors.

ESM Table 3. Estimated number of kidney disease progression events over 3 years with different SGLT2-inhibitor treatment strategies modelled on the study population. Event numbers were estimated using the CKD-PC risk score and the relative treatment effect of SGLT2-inhibitors from previous trial meta-analysis (HR 0.62[1]).

**Strategy**	***N*** **(%) treated with SGLT2-inhibitor**	***N*** **(%) with kidney disease progression event over 3 years**	***N*** **events avoided (% of total avoidable)**	**3-year absolute risk reduction**
Treat none	0 (0%)	1,727 (1.2%)	0 (0%)	-
Treat all	141,500 (100%)	1,075 (0.8%)	652 (100%)	0.46%<br>*(NNT 217)*
Albuminuria threshold ≥3 mg/mmol	25,303 (17.9%)	1,499 (1.1%)	228 (35.0%)	0.90%<br>*(NNT 111)*
$pARR$ threshold ≥0.65%	25,303 (17.9%)	1,474 (1.0%)	253 (38.8%)	1.00%<br>*(NNT 100)*

Abbreviations: $pARR$ predicted 3-year absolute risk reduction with SGLT2-inhibitors, NNT number needed to treat.

ESM Table 4. Baseline characteristics by $pARR$ threshold.

	uACR <3 mg/mmol		uACR 3-30 mg/mmol	
	$pARR$ <0.65% (*n*=108,429)	$pARR$ ≥0.65% (*n*=7768)	$pARR$ <0.65% (*n*=7768)	$pARR$ ≥0.65% (*n*=17,535)
Sociodemographic characteristics				
Age, years	58 ±11	65 ±9	52 ±11	60 ±10
Male sex	64,584 (60%)	3415 (44%)	5059 (65%)	10,918 (62%)
Ethnicity				
White	80,415 (74%)	6305 (81%)	4796 (62%)	12,682 (72%)
South Asian	15,836 (15%)	599 (8%)	1973 (25%)	2732 (16%)
Black	6491 (6%)	556 (7%)	453 (6%)	1194 (7%)
Mixed	1201 (1%)	86 (1%)	111 (1%)	208 (1%)
Other or unknown	4487 (4%)	221 (3%)	435 (6%)	719 (4%)
Index of multiple deprivation, deciles				
1-2	18,729 (17%)	1316 (17%)	1100 (14%)	2650 (15%)
3-4	19,635 (18%)	1412 (18%)	1252 (16%)	2989 (17%)
5-6	20,730 (19%)	1494 (19%)	1410 (18%)	3347 (19%)
7-8	24,171 (22%)	1749 (23%)	1859 (24%)	4125 (24%)
9-10	25,165 (23%)	1796 (23%)	2148 (28%)	4425 (25%)
Laboratory and vital signs measurements				
BMI, kg/m^2	32 ±7	33 ±7	32 ±7	33 ±7
Systolic blood pressure, mmHg	131 ±13	140 ±16	129 ±12	137 ±15
Diastolic blood pressure, mmHg	78 ±8	79 ±10	79 ±8	79 ±10
Total cholesterol, mmol/l	4.4 ±1.1	4.6 ±1.2	4.5 ±1.2	4.5 ±1.2
HbA_{1c}, mmol/mol	73 ±16	92 ±23	72 ±15	82 ±20
HbA_{1c}, %	8.8 ±3.6	10.6 ±4.3	8.7 ±3.5	9.7 ±4.0
eGFR, ml/min/1.73m^2	96 ±14	85 ±14	105 ±12	93 ±15
uACR, mg/mmol	0.8 [0.6, 1.4]	2.3 [1.7, 2.8]	4.1 [3.4, 5.4]	7.8 [4.9, 13.0]

Comorbidities				
Diabetes duration at treatment start, years	6.7 [3.6, 10.8]	8.6 [4.6, 13.3]	5.7 [2.9, 9.5]	8.1 [4.4, 12.6]
Smoking status				
Non-smoker	59,143 (55%)	3167 (41%)	4588 (59%)	7993 (46%)
Ex-smoker	32,803 (30%)	3453 (45%)	1686 (22%)	6130 (35%)
Current smoker	16,483 (15%)	1147 (15%)	1495 (19%)	3412 (20%)
Arterial hypertension	51,941 (48%)	5932 (76%)	2925 (38%)	11,811 (67%)
Atrial fibrillation	1976 (2%)	644 (8%)	28 (0%)	674 (4%)
Previous diabetic keto-acidosis	410 (0%)	55 (1%)	34 (0%)	119 (1%)
Previous mycotic genital infection	12,200 (11%)	1039 (13%)	857 (11%)	2016 (12%)
Hospitalisation in previous year	19,868 (18%)	1695 (22%)	1513 (20%)	3676 (21%)
Medications				
Calendar year at baseline				
2013	12,923 (12%)	903 (12%)	911 (12%)	1926 (11%)
2014	13,893 (13%)	902 (12%)	958 (12%)	2283 (13%)
2015	15,607 (14%)	1155 (15%)	1068 (14%)	2508 (14%)
2016	14,038 (13%)	1083 (14%)	998 (13%)	2233 (13%)
2017	14,163 (13%)	1047 (14%)	1035 (13%)	2324 (13%)
2018	14,759 (14%)	1054 (14%)	1048 (14%)	2447 (14%)
2019	14,906 (14%)	995 (13%)	1166 (15%)	2421 (14%)
2020	8140 (8%)	629 (8%)	584 (8%)	1393 (8%)
Number of current glucose-lowering treatments				
1	11,086 (10%)	811 (10%)	672 (9%)	1482 (9%)
2	63,400 (58%)	3775 (49%)	4767 (61%)	8859 (51%)
3+	33,944 (31%)	3181 (41%)	2329 (30%)	7194 (41%)
On statin	87,872 (81%)	6679 (86%)	5944 (77%)	15,010 (86%)
On insulin	4523 (4%)	1352 (17%)	144 (2%)	1811 (10%)

On ACE-inhibitor or ARB	57,620 (53%)	6394 (82%)	4072 (52%)	14,595 (83%)
Treatment arm				
SGLT2-inhibitor	39,991 (37%)	3087 (40%)	2852 (37%)	7166 (41%)
DPP4-inhibitor/sulfonylurea	68,438 (63%)	4681 (60%)	4916 (63%)	10,369 (59%)

* $pARR$ threshold 0.65%, matching treatment proportion as per treatment strategy based on uACR ≥3 mg/mmol.

Values for continuous variables are mean±SD or median (IQR); those for categorical variables are *n* (%)

Abbreviations: $pARR$ predicted absolute risk reduction, BMI body mass index, eGFR estimated glomerular filtration rate (calculated using the Chronic Kidney Disease Epidemiology Collaboration equation), uACR urinary albumin-creatinine ratio, ACE angiotensin-converting enzyme, ARB angiotensin-II receptor blocker, SGLT2-inhibitors sodium-glucose cotransporter-2 inhibitors, DPP4-inhibitors dipeptidyl peptidase-4 inhibitors.

ESM Figure 1. Flow diagram of study cohort inclusion and exclusion.

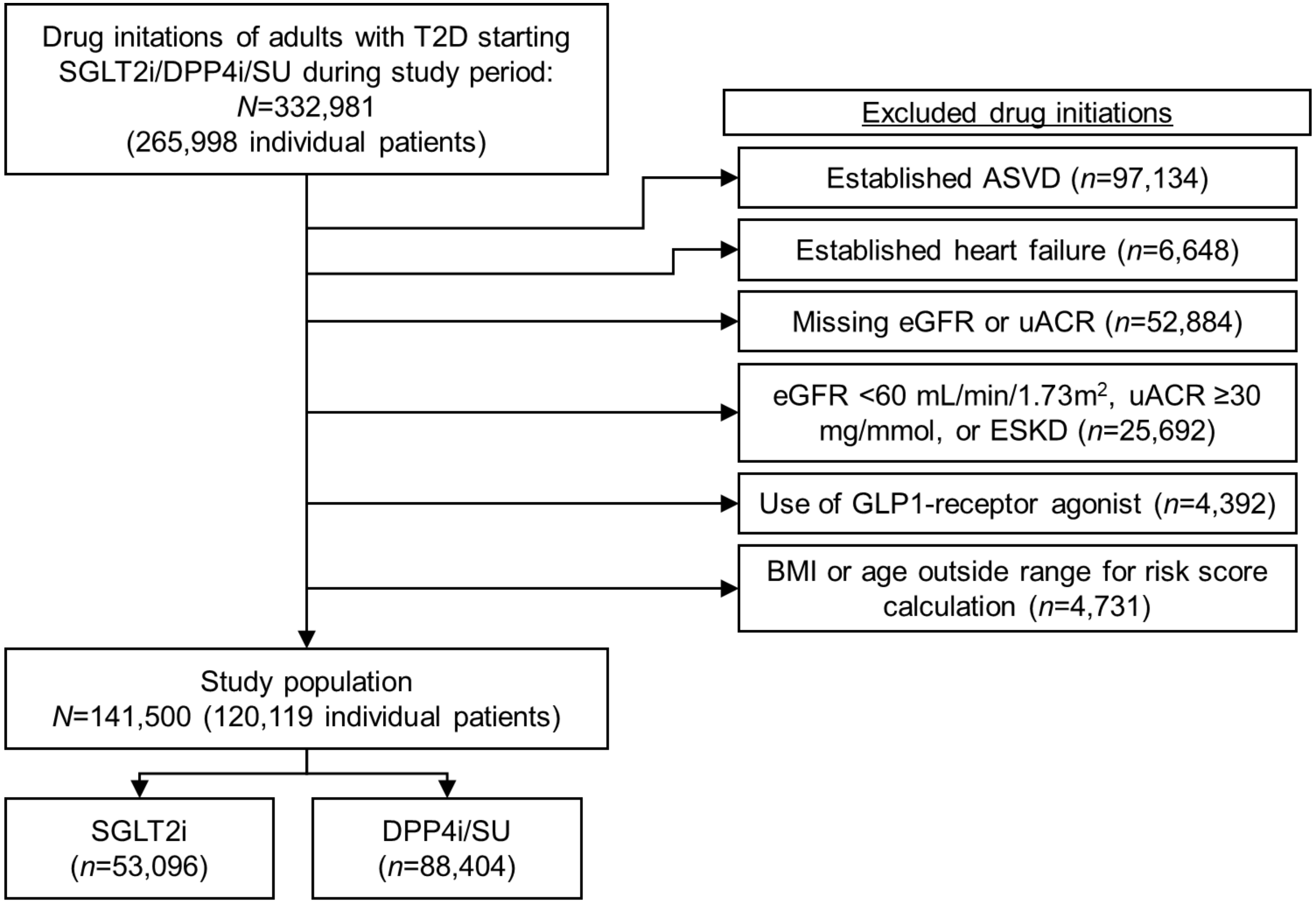

Abbreviations: T2D type 2 diabetes; SGLT2i SGLT2-inhibitors; DPP4i DPP4-inhibitors; SU sulfonylureas; ASVD atherosclerotic vascular disease (ischaemic heart disease or angina, peripheral vascular disease, revascularisation, stroke, or transient ischaemic attack); eGFR estimated glomerular filtration rate; uACR urinary albumin/creatinine ratio; ESKD end-stage kidney disease; BMI body mass index.

ESM Figure 2. Forest plot showing HRs with 95% confidence intervals for kidney disease progression (≥50% decline in eGFR, ESKD, or death due to kidney-related causes) over 3 years with SGLT2-inhibitors from trial meta-analysis (Nuffield Group, 2022) and estimated in this cohort using three analysis approaches. The analysis approaches used in this cohort included multivariable adjustment alone, doubly-robust overlap weighting, and doubly-robust inverse probability of treatment weighting (IPTW). The comparator arms were placebo in the meta-analysis of randomised controlled trials and DPP4-inhibitors/sulfonylureas in this study cohort. Follow-up time was mean 2.1 ±1.0 years in the SGLT2-inhibitor arm, and 2.2 ±1.0 years in the DPP4-inhibitor/sulfonylurea arm.

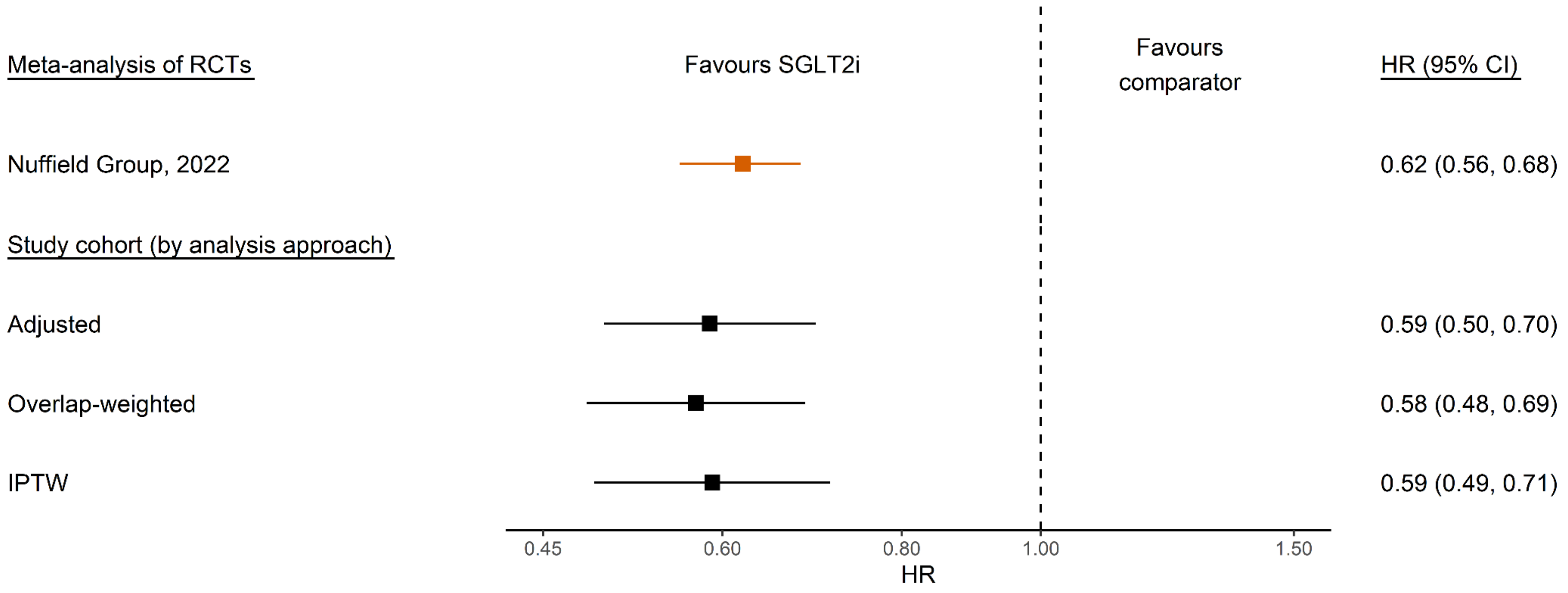

Abbreviations: RCT randomised controlled trial; SGLT2i SGLT2-inhibitor; CI confidence interval; IPTW inverse probability of treatment weighting

ESM Figure 3. Forest plot showing HRs with 95% confidence intervals for kidney disease progression (≥50% decline in eGFR, ESKD, or death due to kidney-related causes) over 3 years with pairwise comparisons between SGLT2-inhibitors, DPP4-inhibitors, and sulfonylureas, using 3 analytical approaches (multivariable adjustment, overlap weighting, and inverse probability of treatment weighting). In these analyses, individuals were also censored if starting a DPP4-inhibitor or sulfonylurea during follow-up whilst in a different treatment arm. They were subsequently included in the respective arm from the same date, allowing inclusion of two non-overlapping follow-up periods for these individuals (*n*=5270 drug episodes of starting a DPP4-inhibitor after an episode of starting a sulfonylurea or vice versa). Of note, in sensitivity analyses comparing SGLT2i-inhibitors to DPP4-inhibitors/sulfonylureas where only a single drug episode per participant was included, the association was similar (overlap-weighted HR 0.52, 95%CI 0.43, 0.61).

SGLT2-inhibitors vs sulfonylureas	Events/subjects (SGLT2i)	Events/subjects (SU)	HR (95% CI)
Adjusted	186/53,096	269/36,854	0.54 (0.43, 0.68)
Overlap-weighted	186/53,096	269/36,854	0.53 (0.40, 0.70)
IPTW	186/53,096	269/36,854	0.53 (0.40, 0.69)

SGLT2-inhibitors vs DPP4-inhibitors	Events/subjects (SGLT2i)	Events/subjects (DPP4i)	HR (95% CI)
Adjusted	186/53,096	395/56,820	0.58 (0.48, 0.70)
Overlap-weighted	186/53,096	395/56,820	0.61 (0.47, 0.79)
IPTW	186/53,096	395/56,820	0.60 (0.48, 0.75)

DPP4-inhibitors vs sulfonylureas	Events/subjects (DPP4i)	Events/subjects (SU)	HR (95% CI)
Adjusted	395/56,820	269/36,854	0.94 (0.79, 1.11)
Overlap-weighted	395/56,820	269/36,854	0.86 (0.71, 1.05)
IPTW	395/56,820	269/36,854	0.89 (0.73, 1.08)

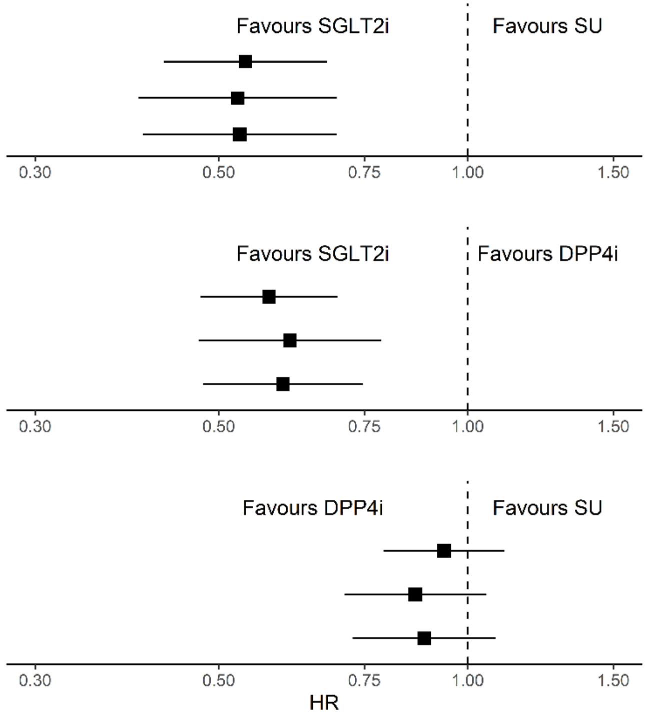

Abbreviations: SGLT2i SGLT2-inhibitors; DPP4i DPP4-inhibitors; SU sulfonylureas; CI confidence interval; IPTW inverse probability of treatment weighting.

ESM Figure 4. Calibration plot of predicted 3-year risk of kidney disease progression (≥50% decline in eGFR, ESKD, or death due to kidney-related causes) using the CKD-PC risk score compared with observed risk, in individuals treated with DPP4-inhibitors/sulfonylureas (*n*=88,404). Observed risk with 95% confidence intervals was calculated using Kaplan-Meier estimates per risk score decile.

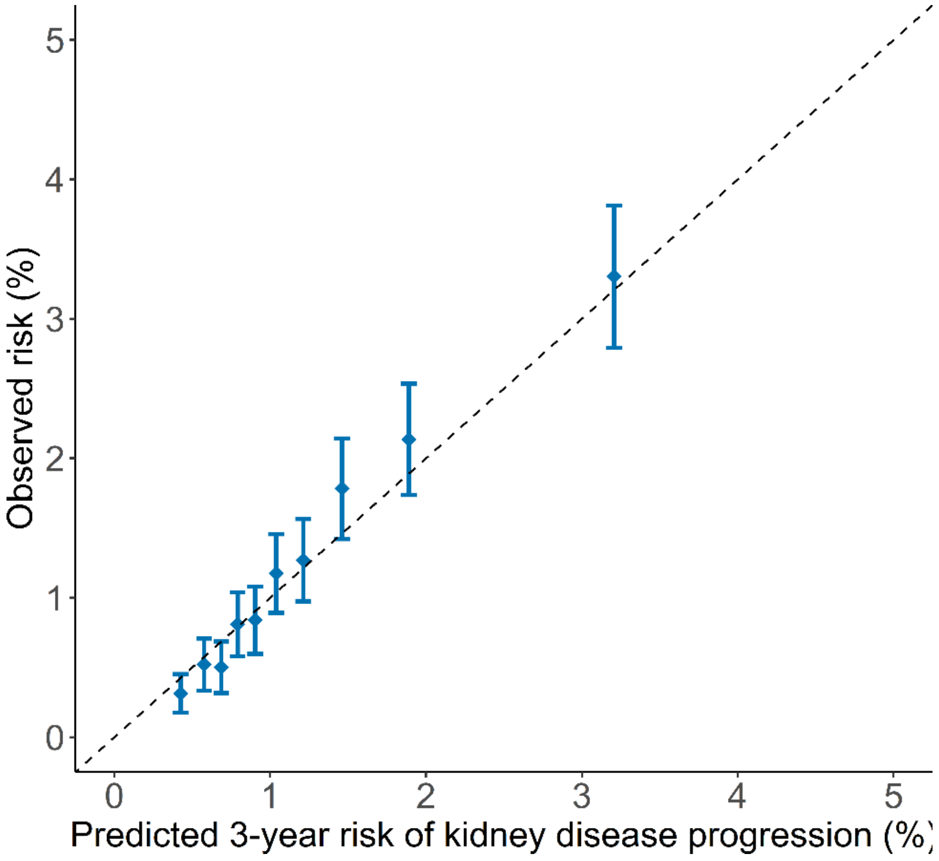

Abbreviations: CKD-PC Chronic Kidney Disease Prognosis Consortium.

ESM Figure 5. Calibration of model-predicted 3-year absolute risk reductions with SGLT2-inhibitor treatment (pARR) compared to counterfactual absolute risk reductions estimated from observed data using doubly robust overlap-weighted Cox models, using only one comparator drug at a time. Median and interquartile range are shown by deciles of pARR. Panel a shows calibration using DPP4-inhibitors as only comparator drug (calibration slope 1.14, 95% CI 1.13-1.16), and panel b shows calibration using sulfonylureas as only comparator drug (calibration slope 1.06, 95%CI 1.04-1.07).

a.

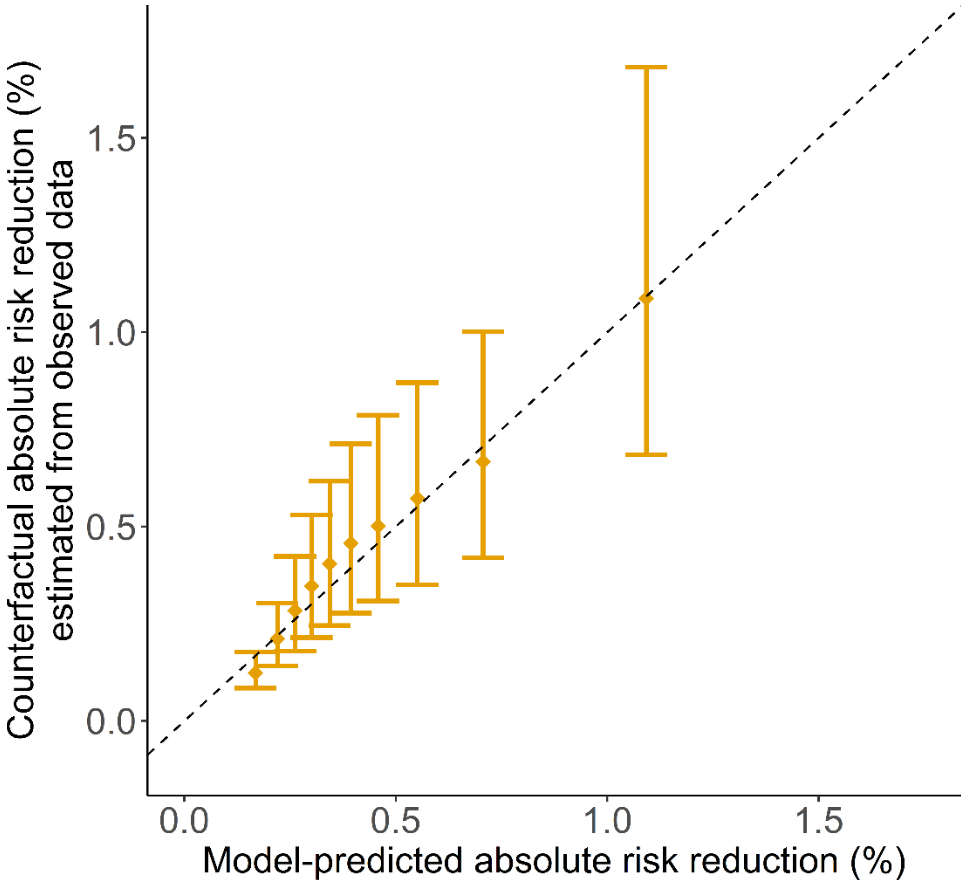

b.

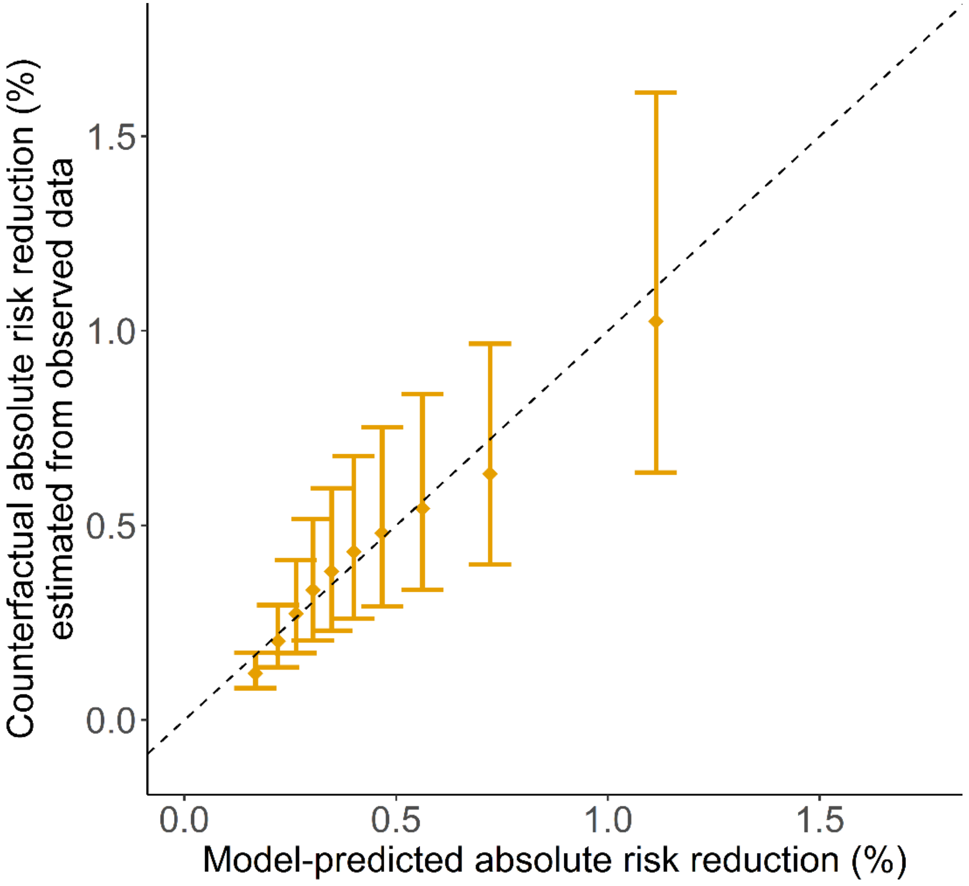

Counterfactual absolute risk reduction (%)
estimated from observed data
1.5
1.0
0.5
0.0
0.0
0.5
1.0
1.5
Model-predicted absolute risk reduction (%)

ESM Figure 6. Decision curve comparing net utility (also referred to as net benefit) of different treatment strategies for targeting individuals at risk of kidney disease progression (≥50% decline in eGFR, ESKD, or death due to kidney-related causes), by risk tolerance (also referred to as threshold probability; of 3-year risk of kidney disease progression). The solid blue line represents the treatment strategy based on uACR ≥3 mg/mmol (as recommended by current guidelines), the dashed orange line a treatment strategy based on $pARR$ ≥0.65%, where a comparable proportion of the population would be treated as under uACR ≥3 mg/mmol, and the solid yellow line represents a $pARR$-based strategy where the $pARR$ threshold varies according to the level of risk tolerance. The black solid line represents a strategy where everyone is treated, whereas the grey solid line represents a strategy where no one is treated. Higher net utility at a given level of risk tolerance indicates greater clinical utility of that strategy.

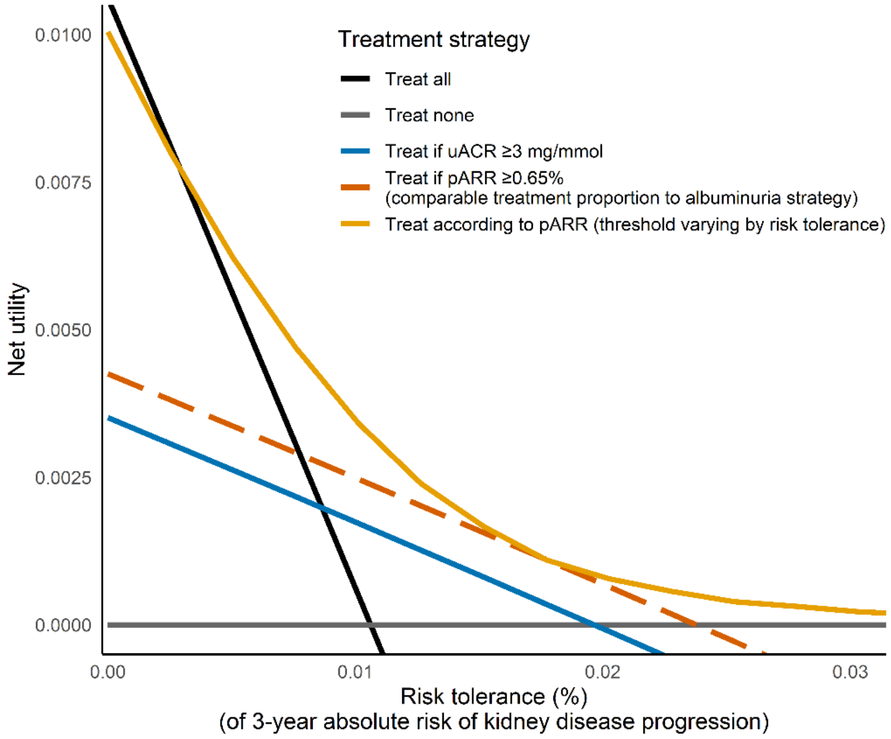

Abbreviations: pARR predicted absolute risk reduction with SGLT2-inhibitor treatment.

ESM Figure 7. Extended 5-year observational analyses of the impact of a treatment strategy based on $pARR$ ≥0.65% vs uACR ≥3 mg/mmol (currently recommended) on cumulative observed absolute risk reductions in kidney disease progression (≥50% decline in eGFR, ESKD, or death due to kidney-related causes). The ≥0.65% $pARR$ threshold was chosen to target a comparable proportion of the population as the ≥3 mg/mmol albuminuria threshold. Observed absolute risk reductions were derived from doubly robust overlap-weighted Cox proportional hazards models smoothed using a loess method.

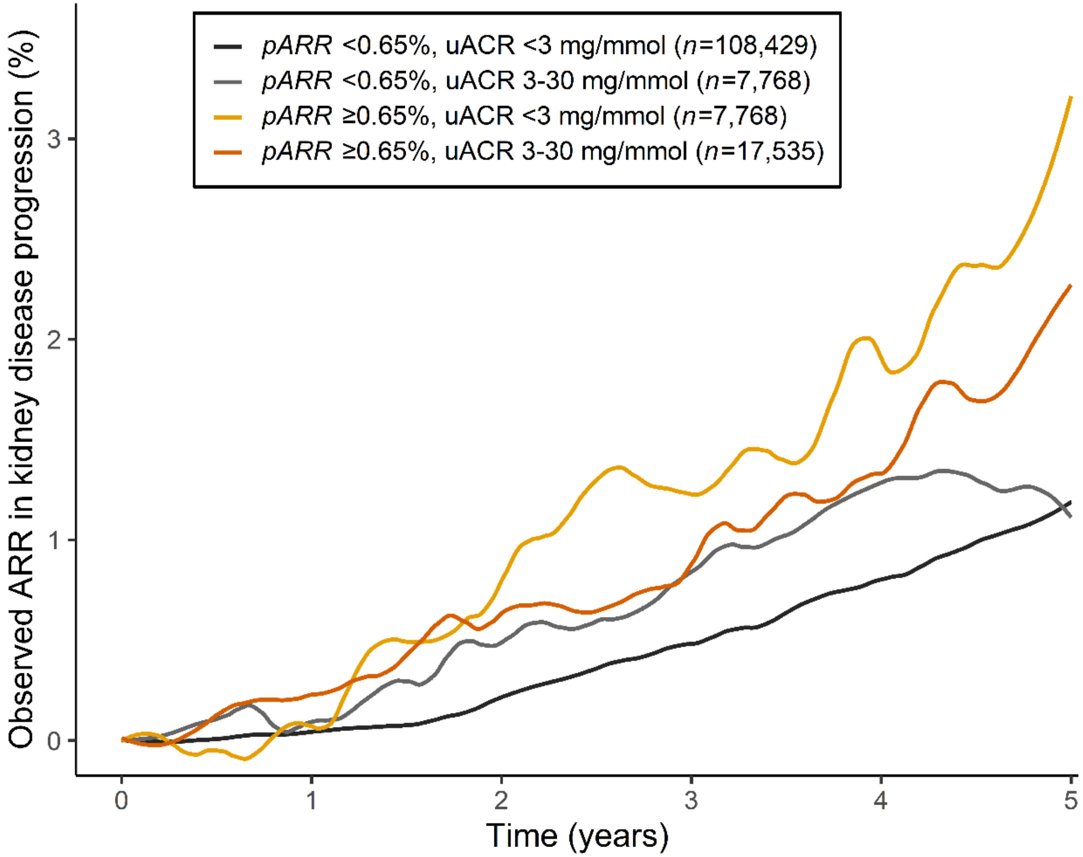

Abbreviations: ARR absolute risk reduction, pARR predicted absolute risk reduction with SGLT2-inhibitor treatment, uACR urinary albumin/creatinine ratio.

ESM Figure 8. Bar chart showing the observed 5-year absolute risk reduction in kidney disease progression (≥50% decline in eGFR, ESKD, or death due to kidney-related causes) with SGLT2-inhibitors in subgroups of individuals by treatment recommendation based on uACR ≥3 mg/mmol (striped vs solid) or predicted benefit ($pARR$; grey vs yellow). The ≥0.65% pARR threshold was chosen to target a comparable proportion of the population to the ≥3 mg/mmol albuminuria threshold. Observed absolute risk reductions were derived from doubly robust overlap-weighted Cox proportional hazards models smoothed using a loess method. Error bars depict 95% confidence intervals.

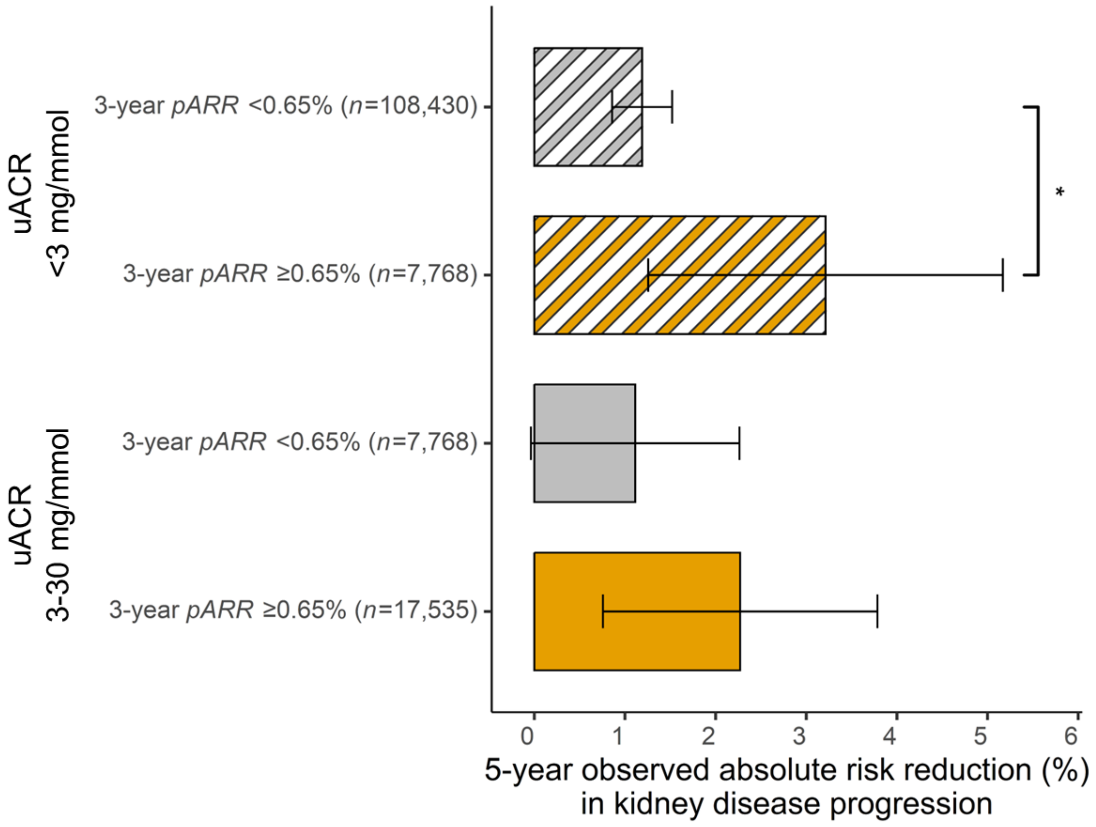

*p=0.05

ESM Figure 9. Forest plots showing HRs with 95% confidence intervals for secondary outcomes over 3 years with SGLT2-inhibitors compared to DPP4-inhibitors/sulfonylureas, stratified by uACR ≥3 mg/mmol (panel A) and $pARR$ ≥0.65% (panel B). Secondary outcomes included a composite outcome of 40% decline in eGFR, ESKD, or death due to kidney-related causes; the composite outcome of 50% decline in eGFR, ESKD, or death due to kidney-related causes (5 years rather than 3 years); progression to significant albuminuria (≥30 mg/mmol); diabetic keto-acidosis; amputation; and mycotic genital infection. Hazard ratios were derived from doubly robust overlap-weighted analyses. Baseline characteristics by $pARR$ are shown in ESM Table 4.

a.

	Events/subjects (SGLT2i)	(DPP4i/SU)	HR (95% CI)
40% eGFR decline / ESKD			
uACR <3 mg/mmol	357/43,078	1,039/73,119	0.68 (0.59, 0.79)
uACR 3-30 mg/mmol	165/10,018	415/15,285	0.66 (0.54, 0.82)
50% eGFR decline / ESKD (5 years)			
uACR <3 mg/mmol	208/43,078	837/73,119	0.53 (0.44, 0.63)
uACR 3-30 mg/mmol	131/10,018	369/15,285	0.65 (0.52, 0.83)
Progression to uACR ≥30 mg/mmol			
uACR <3 mg/mmol	23/43,078	64/73,119	0.83 (0.48, 1.43)
uACR 3-30 mg/mmol	86/10,018	348/15,285	0.53 (0.40, 0.70)
Diabetic keto-acidosis			
uACR <3 mg/mmol	178/43,078	155/73,119	1.80 (1.37, 2.36)
uACR 3-30 mg/mmol	62/10,018	38/15,285	2.39 (1.41, 4.05)
Amputation			
uACR <3 mg/mmol	51/43,078	64/73,119	1.26 (0.79, 1.99)
uACR 3-30 mg/mmol	30/10,018	38/15,285	1.13 (0.64, 2.02)
Mycotic genital infection			
uACR <3 mg/mmol	3,348/43,078	1,992/73,119	3.10 (2.90, 3.32)
uACR 3-30 mg/mmol	709/10,018	451/15,285	2.76 (2.39, 3.19)

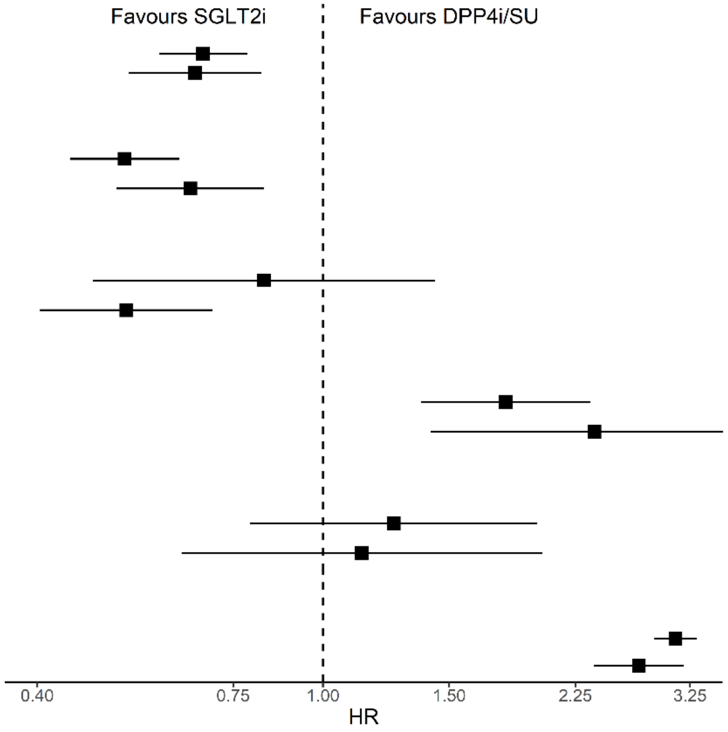

b.

	Events/subjects (SGLT2i)	(DPP4i/SU)	HR (95% CI)
40% eGFR decline / ESKD			
pARR <0.65%	326/42,843	934/73,355	0.69 (0.60, 0.80)
pARR ≥0.65%	196/10,253	520/15,049	0.65 (0.54, 0.79)
50% eGFR decline / ESKD (5 years)			
pARR <0.65%	182/42,843	755/73,355	0.52 (0.43, 0.63)
pARR ≥0.65%	157/10,253	451/15,049	0.65 (0.52, 0.80)
Progression to uACR ≥30 mg/mmol			
pARR <0.65%	35/42,843	125/73,355	0.61 (0.40, 0.93)
pARR ≥0.65%	74/10,253	287/15,049	0.54 (0.40, 0.73)
Diabetic keto-acidosis			
pARR <0.65%	168/42,843	141/73,355	2.13 (1.60, 2.84)
pARR ≥0.65%	72/10,253	52/15,049	1.53 (0.99, 2.38)
Amputation			
pARR <0.65%	43/42,843	65/73,355	1.12 (0.69, 1.80)
pARR ≥0.65%	38/10,253	37/15,049	1.38 (0.79, 2.39)
Mycotic genital infection			
pARR <0.65%	3,321/42,843	2,007/73,355	3.16 (2.95, 3.38)
pARR ≥0.65%	736/10,253	436/15,049	2.55 (2.20, 2.95)

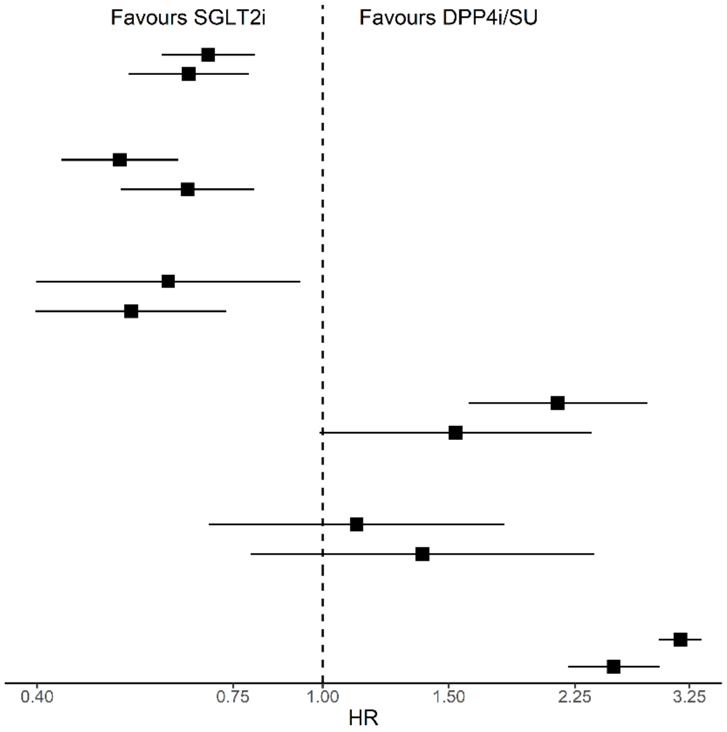

Abbreviations: SGLT2i SGLT2-inhibitors; DPP4i/SU DPP4-inhibitors/sulfonylureas; CI confidence interval; pARR predicted absolute risk reduction with SGLT2-inhibitor treatment.

MASTERMIND consortium

Prof Andrew Hattersley[1], Prof Ewan Pearson[2], Dr Angus Jones[1], Dr Beverley Shields[1], Dr John Dennis[1], Dr Lauren Rodgers[1], Prof William Henley[1], Prof Timothy McDonald[1], Prof Michael Weedon[1], Prof Nicky Britten[1], Catherine Angwin[1], Dr Naveed Sattar[3], Dr Robert Lindsay[3], Prof Christopher Jennison[4], Prof Mark Walker[5], Prof Kennedy Cruickshank[6], Dr Salim Janmohamed[7], Prof Christopher Hyde[1], Prof Rury Holman[8], Prof Andrew Farmer[8], Prof Alastair Gray[8], Prof Stephen Gough[8], Dr Olorunsola Agbaje[8], Dr Trevelyan McKinley[1], Dr Sebastian Vollmer[9], Dr Bilal Mateen[7], Prof William Hamilton[1], Dr Katie G. Young[1], Dr Pedro Cardoso[1,] Dr Laura Güdemann[1], Dr Thijs Jansz[1]

[1] University of Exeter

[2] University of Dundee

[3] University of Glasgow

[4] University of Bath

[5] University of Newcastle

[6] Kings College London

[7] University College London

[8] University of Oxford

[9] University of Kaiserslautern
